# Correction of immunosuppression in aged septic rats by human ghrelin and growth hormone through the vagus nerve-dependent inhibition of TGF-β production

**DOI:** 10.1186/s10020-020-00195-x

**Published:** 2020-07-16

**Authors:** Mian Zhou, Monowar Aziz, Mahendar Ochani, Ping Wang

**Affiliations:** 1 Center for Immunology and Inflammation, The Feinstein Institutes for Medical Research, Manhasset, New York USA; 2 Departments of Surgery and Molecular Medicine, Donald and Barbara Zucker School of Medicine at Hofstra/Northwell, Manhasset, New York USA

**Keywords:** Ghrelin, Aging, Sepsis, Immunosuppression, Vagus nerve

## Abstract

**Background:**

Co-administration of human ghrelin and growth hormone (GH) reverse immunosuppression in septic aged animals, but the mechanism remains elusive. Here, we hypothesize that ghrelin and GH co-treatment restores the immune response in aged septic rats by inhibiting the production of transforming growth factor-β (TGF-β), an immunoregulatory cytokine, through the vagus nerve.

**Methods:**

Male aged Fischer rats (22–23-month-old) were made septic by cecal ligation and puncture (CLP) with or without dissecting the vagus nerve (vagotomy). Human ghrelin and GH or vehicle (PBS) were administrated subcutaneously at 5 h post CLP. After 20 h of CLP, serum and spleens were harvested.

**Results:**

Serum TGF-β levels were increased in septic aged rats, while ghrelin and GH treatment significantly reduced its levels. Expression of TGF-β in the spleen was upregulated after sepsis, while ghrelin and GH treatment significantly inhibited its expression. TNF-α and IL-6 levels were significantly reduced after ex vivo LPS stimulation of splenocytes from rats that underwent CLP compared to sham rats; while these levels were significantly higher in splenocytes from ghrelin and GH-treated CLP rats compared to vehicle-treated CLP rats. Ghrelin and GH treatment reduced program death receptor-1 (PD-1) expression, increased human leukocyte antigen-DR (HLA-DR) expression, attenuated lymphopenia, and cleaved caspase-3 levels in the spleen of septic aged rats. Vagotomy diminished the beneficial effects of ghrelin and GH treatment in septic rats. In vitro, the addition of ghrelin, GH, or ghrelin and GH together had no effect on restoring immune response in splenocytes from CLP rats following LPS stimulation, indicating the requirement of the vagus nerve for ghrelin and GH’s effect.

**Conclusions:**

Ghrelin and GH attenuate immunosuppression in aged septic rats through the vagus nerve-dependent inhibition of TGF-β production.

## Introduction

Sepsis is a life-threatening condition that arises due to a dysregulated immune response to infection leading to excessive inflammation and organ injury (Singer et al. 2016). Although an uncontrolled inflammatory syndrome is the prevailing theory for sepsis, subsequent development of an immunosuppressive phenomenon is increasingly being recognized (Delano and Ward 2016; Hotchkiss et al. 2013). Studies with human septic patients demonstrate that immunosuppression is the predominant cause of morbidity and mortality in sepsis (Hotchkiss et al. 2013; Boomer et al. 2011). Immunosuppression is commonly encountered in elderly patients that develop sepsis, resulting in impaired efficacy in fighting against invading pathogens (Hotchkiss et al. 2013; Boomer et al. 2011). Persistent inflammation, T cell exhaustion, and lymphocytopenia are observed in elderly patients after sepsis (Inoue et al. 2014). Those patients are susceptible to secondary infection with lower survival rates compared to young septic patients (Inoue et al. 2014). Studies have shown that severe sepsis and septic shock are mainly observed in elderly patients (> 65 years of age), giving rise to significantly higher mortality rate of about 80% (Boomer et al. 2011; Martin et al. 2006; Opal et al. 2005; Hepper et al. 2013). In-depth understanding of the pathophysiology and the development of novel therapeutics are urgently needed to protect the elderly from sepsis.

The pleiotropic cytokine transforming growth factor-β (TGF-β) is a key regulator of the immune response to infection (Yoshimura et al. 2010; Santarpia et al. 2015). TGF-β is produced by a wide number of cells including leukocytes as well as epithelial cells (Taylor 2009). It controls the differentiation, proliferation and activation of immune cells (Taylor 2009; Weehuizen et al. 2012). There are 3 isoforms of TGF-β that have been identified in mammals, namely TGF-β1, -β2 and -β3. These isoforms of TGF-β have similar biological function but are expressed in different tissues (Govinden and Bhoola 2003). Among these three isoforms, TGF-β1 is predominantly expressed by the cells of the immune system (Govinden and Bhoola 2003). TGF-β has multiple immunosuppressive properties as evidenced by the fact that TGF-β knockout mice develop multi-organ autoimmune inflammatory disease and die shortly after birth (Govinden and Bhoola 2003). Although the levels of TGF-β are elevated in patients with sepsis (Marie et al. 1996), its role in inducing immunosuppression is not fully understood.

Ghrelin is a small peptide predominantly produced by the gastrointestinal tract (Collden et al. 2017). Ghrelin binds to the growth hormone secretagogue receptor (GHSR)-1a to promote the release of growth hormone (GH) (Arvat et al. 2000). Beyond its metabolic function, ghrelin has anti-inflammatory and anti-apoptotic functions as well (Wu and Kral 2004; Wu et al. 2007a; Gonzalez-Rey et al. 2006; Zhou et al. 2017). Our previous studies showed that co-treatment of ghrelin and GH mitigated organ injury and improved survival in aged rats after sepsis (Wu et al. 2009a; Yang et al. 2016). Even though we recently demonstrated that the combined treatment with ghrelin and GH (GG) were able to reverse immunosuppression in aged septic rats, the underlying mechanism involving the potential role of TGF-β in GG-mediated restoration of immune response had not been studied (Zhou et al. 2017). We therefore investigated whether the effect of GG on restoring immune response in aged animals with sepsis was mediated through the modulation of TGF-β production. We further aimed to determine the contribution of the vagus nerve for GG-mediated restoration of immune function in aged septic rats.

## Material and methods

### Animal model of sepsis and vagotomy

Aged male Fisher rats (22–23 month-old) were obtained from Charles River Laboratories via the National Institute for Aging, National Institutes of Health (NIH). Animals were housed in a temperature-controlled room with a 12 h light-dark cycle and fed a standard Purina rat chow diet. Rats were allowed to acclimate to the environment for at least 5 days before being used for experiments.

Sepsis was induced in aged rats by cecal ligation and puncture (CLP) (Yang et al. 2016). Briefly, rats were anesthetized by 2% isoflurane inhalation. The abdomen was shaved and cleaned with iodine and alcohol solution, and a 2-cm midline incision was made. The cecum was then exposed and 70% of its length was ligated using 4–0 silk suture distal to the ileocecal valve. The cecum was punctured twice with an 18-G needle and a small amount of feces was extruded. The cecum was then returned to the abdominal cavity, the abdominal incision was closed in layers, and the animals were resuscitated with 30 ml/kg body weight normal saline subcutaneously. In sham rats, the same surgical procedure was performed with the exception that their cecum was not ligated or punctured.

Vagotomy procedure was performed on a group of rats at the time of CLP as reported previously (Wu et al. 2007b; Williams et al. 2003). The trunks of the subdiaphragmatic vagus nerve were transected. Briefly, the dorsal and ventral branches of the vagus nerve were dissected and each branch of the nerve was tied with surgical sutures at 2 points separated by about 1 cm, and then severed between the sutures. After surgery, animals were allowed to eat and drink food and water, respectively.

### Administration of human ghrelin and human growth hormone into aged septic rats

Human ghrelin (Phoenix Pharmaceuticals, Belmont, CA) and human GH (ProSpec, Ness Ziona, Israel) were dissolved in normal saline. A 500 μl mixture of human ghrelin and GH (GG) was prepared and injected subcutaneously to CLP animals at 5 h after CLP. Each GG treated animal received 80 nmol/kg human ghrelin and 50 μg/kg human GH in a single bolus dose.

### Splenocyte isolation and stimulation

Spleens were harvested at 20 h after CLP or sham-operation and homogenized by gentle grinding between frosted glass slides, followed by passage through a 70-μm cell strainer to obtain single cell suspensions (BD Biosciences, San Jose, CA). The suspension was centrifuged at 450×g for 5 min and the pellet was suspended into 44% percoll solution (Sigma, St. Louis, MO), then careflly overlaid on top of 66% percoll solution, centrifuged at 800×g for 30 min at room temperature. After density gradient centrifuge, leukocytes formed a white fluffy ring at the interface between 44% percoll and 66% percoll and were collected into RPMI-1640 medium (Life Technologies, Grand Island, NY) containing 10% heat-inactivated fetal bovine serum, 2 mM L-glutamine, 100 U/ml penicillin, 100 μg/ml streptomycin,10 mM HEPES and 0.5 μM 2-mercaptaethanol. Cell viability was greater than 90%. To evaluate the immune responses of these cells, 2 × 10^6^ cells were plated into a 24-well plate and stimulated with LPS (100 ng/ml, Sigma, St. Louis, MO) for 5 h. The released proinflammatory cytokines in the culture medium were measured by enzyme-linked immunosorbent assay (ELISA).

### Measurement of cytokines and analysis of lymphocyte, monocyte, and basophil numbers

Cytokine levels were quantified using ELISA kits specific for rat TNF-α, IL-6 (BD Biosciences) and TGF-β1 (eBioscience, San Diego, CA). All measurements were performed according to manufacturer’s instructions. Lymphocyte, monocyte, and basophil numbers in the blood were analyzed by using a Cell-DYN 3700 analyzer (Abbott, Abbott Park, IL).

### Western blot analysis

Spleen tissues were homogenized and lysed in RIPA buffer, 10 mM Tris buffer, pH 7.5 containing 0.1% Triton-X 100, 1 mM EDTA, 1 mM EGTA, protease inhibitor tablet (Thermo Fisher, Waltham, MA), phosphatase inhibitor tablet (Thermo Fisher). Protein concentration was determined by Bio-Rad DC protein assay kit (Bio-Rad, Hercules, CA). Tissue lysates were electrophoresed on 4–12% NuPAGE Bis-Tris Gel (Life Technologies) and transferred to nitrocellulose membranes. The membranes were then blocked with 0.1% casein in Tris buffer saline and incubated with anti-TGF-β (Proteintech, Chicago, IL), -cleaved caspase-3 (Cell Signaling Technologies, Danvers, MA), and -β-actin (Sigma) Abs overnight at 4 °C. After washing, membranes were incubated with infrared dye-labeled respective secondary Abs (LI-COR, Lincoln, NE). The Odyssey infrared image system (LI-COR) was used to analyze the target bands and intensities of bands were qualified using Image Studio Lite software (LI-COR).

### Immunohistochemical analysis

Spleen tissues were fixed in 10% buffered formalin solution for 2 days and processed for paraffin sections using standard histology procedures. Paraffin sections were dewaxed in 100% xylenes and rehydrated in series concentrations of alcohols of 100, 95, 70% to water. Then, antigen retrieval procedure was performed using antigen unmasking solution (Vector Labs, Burlingame, CA). Slides were then incubated with rabbit anti-rat PD-1 (1:50 dilution, Abcam, Cambridge, MA) and rabbit anti-rat HLA-DR (1:50 dilution, Proteintech, Chicago, IL) antibodies overnight at 4 °C. Slides were washed with Tris-buffered saline containing 0.02% Triton x-100 and further reacted with biotinylated anti-rabbit IgG (Vector Labs). Then, the slides were incubated with peroxidase conjugated avidin followed by reaction with substrate, DAB (Vector Labs). Slides were counterstained with hematoxylin and evaluated using a Nikon microscope.

### Statistical analysis

All data are expressed as mean ± SEM and compared by one-way ANOVA and Student-Newman-Keuls (SNK) test and Student’s t-test. Differences in values were considered significant when *P* < 0.05.

## Results

### Combined treatment with ghrelin and GH reduces TGF-β production in aged septic rats through the vagus nerve

We found that the protein levels of TGF-β1 in the serum and spleen were increased by 2.1 and 2.0 folds, respectively in aged rats at 20 h after CLP as compared to the sham-operated animals **(**Fig. 1a, b**)**. Treatment with GG significantly lowered the levels of TGF-β in the serum and spleen of septic aged rats by 52 and 25%, respectively at 20 h after CLP **(**Fig. 1a, b**)**. On the other hand, vagotomy eliminated the inhibitory effect of GG on serum and spleen levels of TGF-β in septic aged rats **(**Fig. 1a, b**)**. Thus, GG modulates the production of TGF-β in the serum and spleen through the vagus nerve. Of note, in the current study, we did not include CLP and vagotomy without GG group, because our previous study showed that vagotomy had no effect on cytokine production in septic rats compared to non-vagotomized animals (Wu et al. 2007b). As such, the production of TGF-β in CLP and vagotomy without GG group may not be altered compared to the CLP only group.

**Fig. 1:**
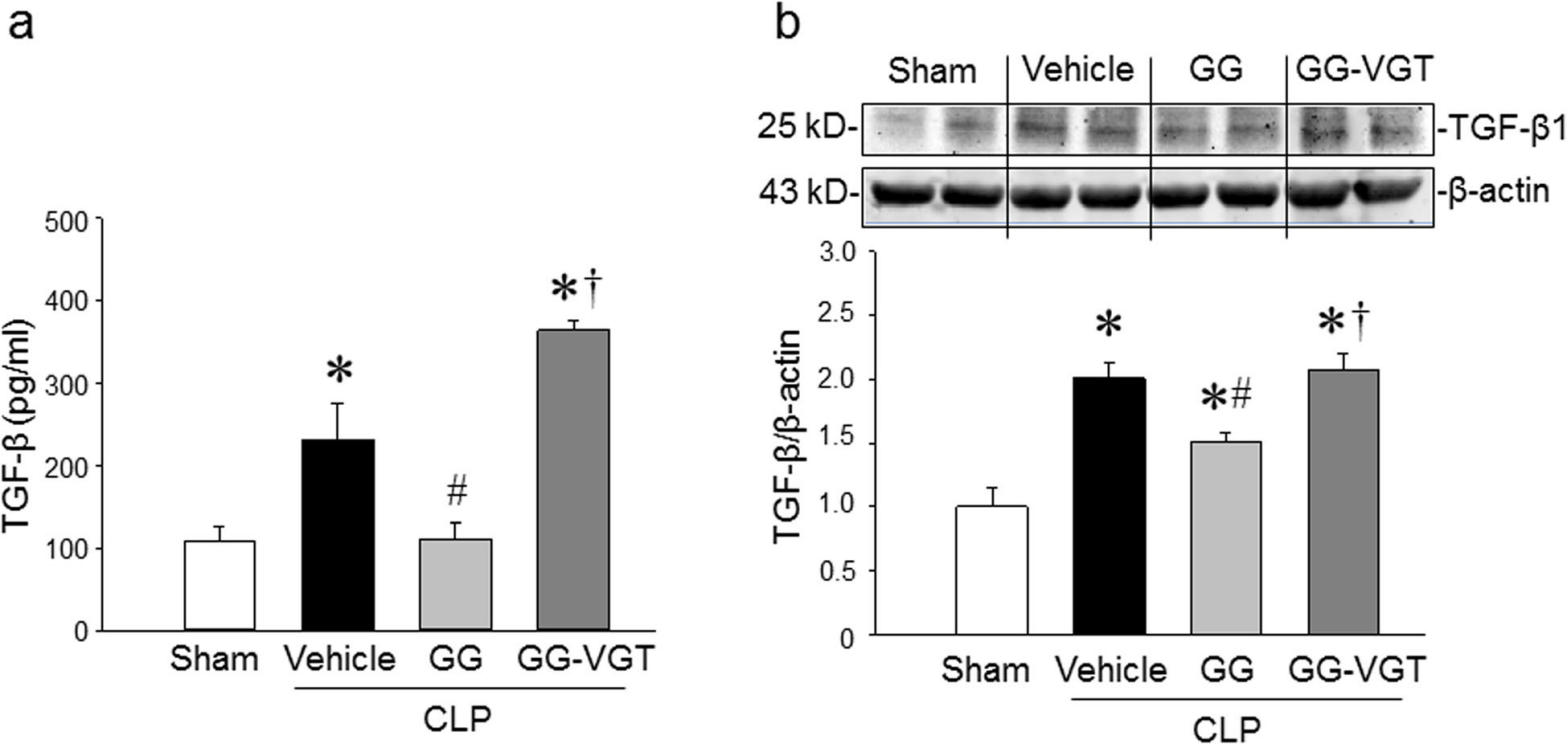
Ghrelin and GH in combination downregulates TGF-β in aged rats with sepsis. Rats were subjected to sham or CLP operation and treated with vehicle (normal saline) or GG (ghrelin 80 nmol/kg, GH 50 μg/kg) at 5 h after CLP. In an additional group of rats, vagotomy was performed at the time of CLP and treated with GG at 5 h after CLP. Serum and spleens were collected at 20 h after CLP. **a** The levels of TGF-β in the serum was measured by ELISA. Data are expressed as mean ± SEM (*n* = 4–7 rats/group). **b** The expression of TGF-β in the spleen were determined by western blotting. The representative western blot images are shown. Sham was normalized as 1 in western blot analysis. Data are expressed as mean ± SEM (*n* = 4–5 rats/group). *P < 0.05 vs. sham; ^#^*P* < 0.05 vs. CLP with vehicle treatment; ^†^*P* < 0.05 vs. CLP with GG treatment. CLP, cecal ligation and puncture; GG, ghrelin and growth hormone in combination; VGT, vagotomy

### Combined treatment of ghrelin and GH improves the immune response of splenocytes from aged septic rats

The immune response of aged rats at 20 h after CLP was evaluated by the release of cytokines from isolated splenocytes in response to ex vivo LPS stimulation. After 5 h of incubation with LPS, the levels of TNF-α and IL-6 in the culture supernatants of splenocytes from sham animals were 520 ± 10 and 218 ± 9 pg/ml, respectively, while the levels of these cytokines were dramatically lower in vehicle-treated septic aged rats (Fig. 2a, b). On the other hand, the culture supernatants of splenocytes isolated from GG-treated aged septic rats showed significantly increased levels of TNF-α and IL-6 at the levels of 287 ± 10 and 149 ± 11 pg/ml, respectively, in response to ex vivo LPS-stimulation as compared to the vehicle treated rats (Fig. 2a, b). Interestingly, the GG-treated vagotomized animals were not able to restore the levels of TNF-α and IL-6 in the culture supernatants after ex vivo stimulation with LPS (Fig. 2a, b). These results demonstrate the restoration of the immune response of aged septic animals by GG treatment was mediated through the vagus nerve.

**Fig. 2:**
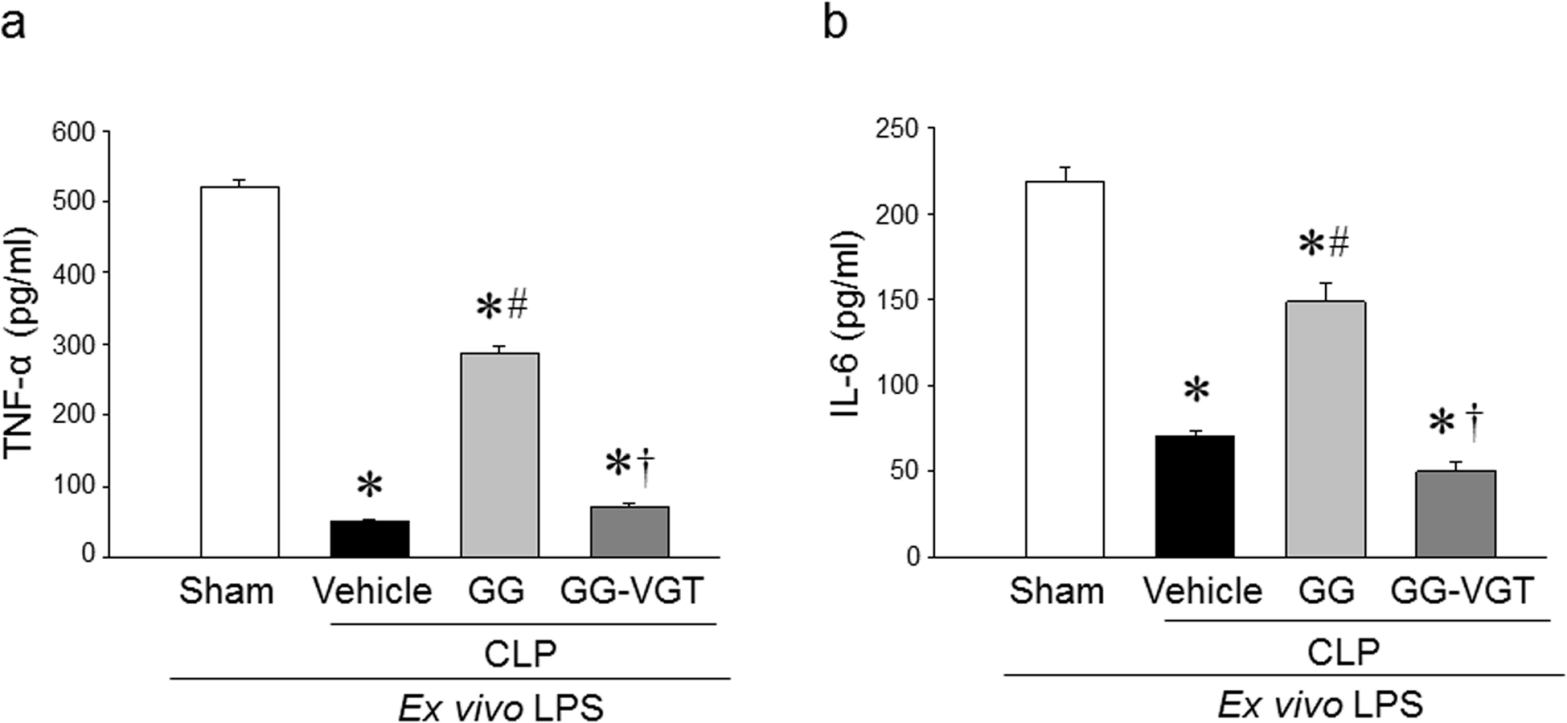
Ghrelin and GH in combination restores immune response in aged septic rats. Rats were subjected to sham or CLP operation and treated with vehicle (normal saline) or GG (ghrelin 80 nmol/kg, GH 50 μg/kg) at 5 h after CLP. In an additional group of rats, vagotomy was performed at the time of CLP and treated with GG at 5 h after CLP. Spleens were harvested at 20 h after CLP. Splenocytes were isolated and stimulated with LPS (100 ng/ml) for 5 h. The release of (**a**) TNF-α and (**b**) IL-6 in the medium were measured by ELISA. Data are expressed as mean ± SEM (*n* = 4–5 rats/group). *P < 0.05 vs. sham; ^#^*P* < 0.05 vs. CLP with vehicle treatment; ^†^*P* < 0.05 vs. CLP with GG treatment

### Co-treatment with ghrelin and GH corrects lymphopenia and reduces cleaved caspase-3 levels in the spleen

The loss of lymphocytes, often resulting in a diminished capacity of the host to fight against pathogens, is a primary feature of immune suppression in critically ill septic patients (Hotchkiss et al. 2013), resulting in them developing secondary infections (Hotchkiss et al. 2013; Boomer et al. 2011; Zhou et al. 2017). Hematological analysis of the blood from sham and septic aged rats showed that lymphocyte count and percentage were dramatically reduced from 2.57 × 10^3^/μl and 37.8% in sham, respectively, to 0.52 × 10^3^/μl and 14.4% in aged septic rats, respectively (Fig. 3a, b). GG treatment significantly increased lymphocyte count and percentage to 1.05 × 10^3^/μl and 27.0%, respectively, while vagotomy in septic rats diminished the GG’s effect on increasing the count and percentage of lymphocytes in the blood (Fig. 3a, b). The levels of cleaved caspase-3, an apoptosis inducing protein, were upregulated in the spleen of septic aged rats at 20 h after CLP (Fig. 3c), suggesting that apoptosis was involved in the loss of lymphocytes in sepsis. The GG treatment significantly downregulated the expression of cleaved-caspase-3 in the spleen of septic aged animals compared to vehicle treated septic aged rats (Fig. 3c). By contrast, the beneficial effects of GG were diminished in vagotomized animals, suggesting the action of GG for reversing lymphocytopenia and inhibiting the production of cleaved caspase-3 protein was mediated through the vagus nerve dependent mechanism (Fig. 3c).

**Fig. 3:**
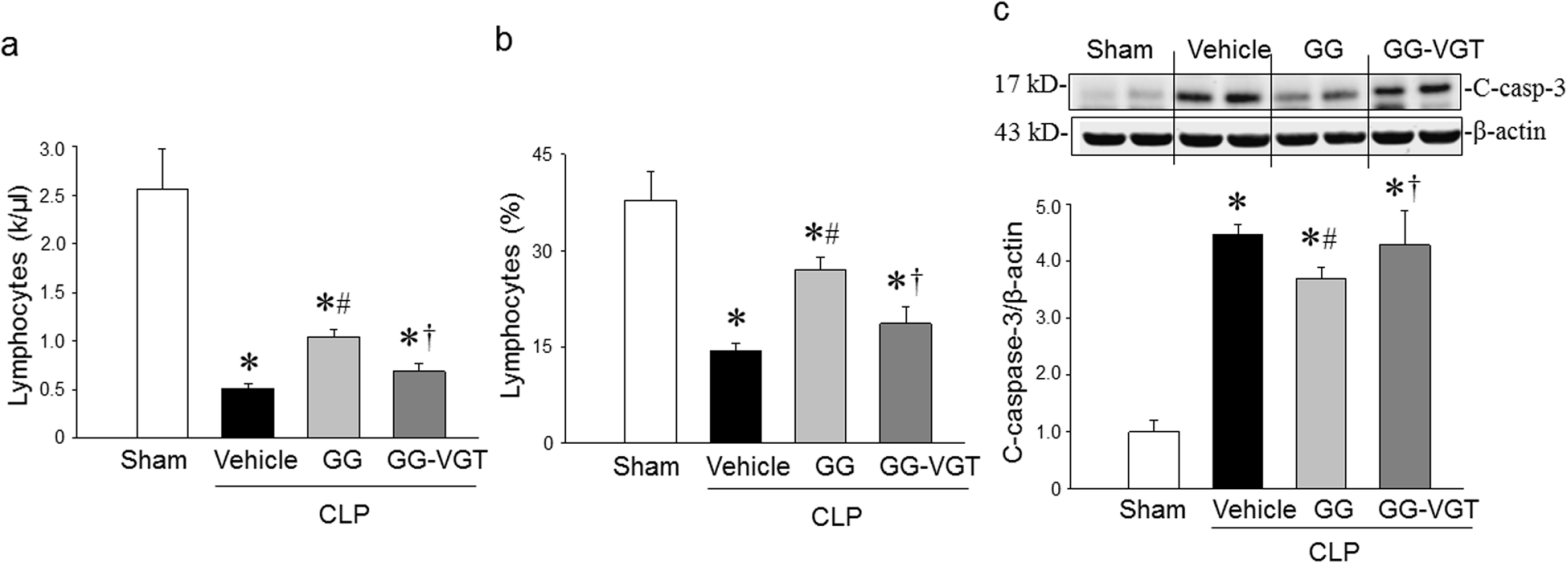
Ghrelin and GH in combination attenuate lymphopenia in aged rats with sepsis in a vagus nerve dependent manner. Rats were subjected to sham or CLP operation and treated with vehicle (normal saline) or GG (ghrelin 80 nmol/kg, GH 50 μg/kg) at 5 h after CLP. In an additional group of rats, vagotomy was performed at the time of CLP and treated with GG at 5 h after CLP. Blood and spleens were collected at 20 h after CLP. **a, b** Circulating lymphocytes were measured using a hematology analyzer. Data are expressed as mean ± SEM (*n* = 4–6 rats/group). **c** The levels of cleaved caspase-3 in the spleen were determined by western blotting. The representative western blot images are shown. Sham was normalized as 1 in western blot analysis. Data are expressed as mean ± SEM (*n* = 4–5 rats/group). *P < 0.05 vs. sham; ^#^*P* < 0.05 vs. CLP with vehicle treatment; ^†^*P* < 0.05 vs. CLP with GG treatment

### Co-treatment with ghrelin and GH corrects monocytosis and basophilia in sepsis

Monocytes are an essential part of the cellular innate immune system and play an important role in host defense against pathogens (Serbina and Pamer 2006). Recruitment of monocytes is essential for effective control and clearance of bacterial infection, but monocytes can also contribute to tissue destruction during some infectious and inflammatory diseases (Serbina and Pamer 2006). Peripheral monocytosis is associated with respiratory symptoms, later infection, and higher mortality in patients admitted to the emergency room (Hensel et al. 2017). Hematological analysis of the blood from sham and septic aged rats showed that monocyte count and percentage markedly increased from 0.43 × 10^3^/μl and 9.1% in sham, respectively, to 0.75 × 10^3^/μl and 18.9% in aged septic rats, respectively (Fig. 4a, b). GG treatment significantly reduced monocytes count and percentage to 0.57 × 10^3^/μl and 13.7%, respectively, while vagotomy in septic rats diminished the GG’s effect on reducing the count and percentage of monocytes in the blood (Fig. 4a, b). Interestingly, similar results were obtained from circulating basophils. The blood from sham and septic aged rats showed that basophil count and percentage dramatically increased from 0.18 × 10^3^/μl and 3.1% in sham, respectively, to 0.36 × 10^3^/μl and 10.8% in aged septic rats, respectively (Fig. 4c, d). GG treatment significantly decreased basophil count and percentage to 0.227 × 10^3^/μl and 6.3%, respectively, while vagotomy in septic rats diminished the GG’s effect on decreasing the count and percentage of basophils in the blood (Fig. 4c, d).

**Fig. 4:**
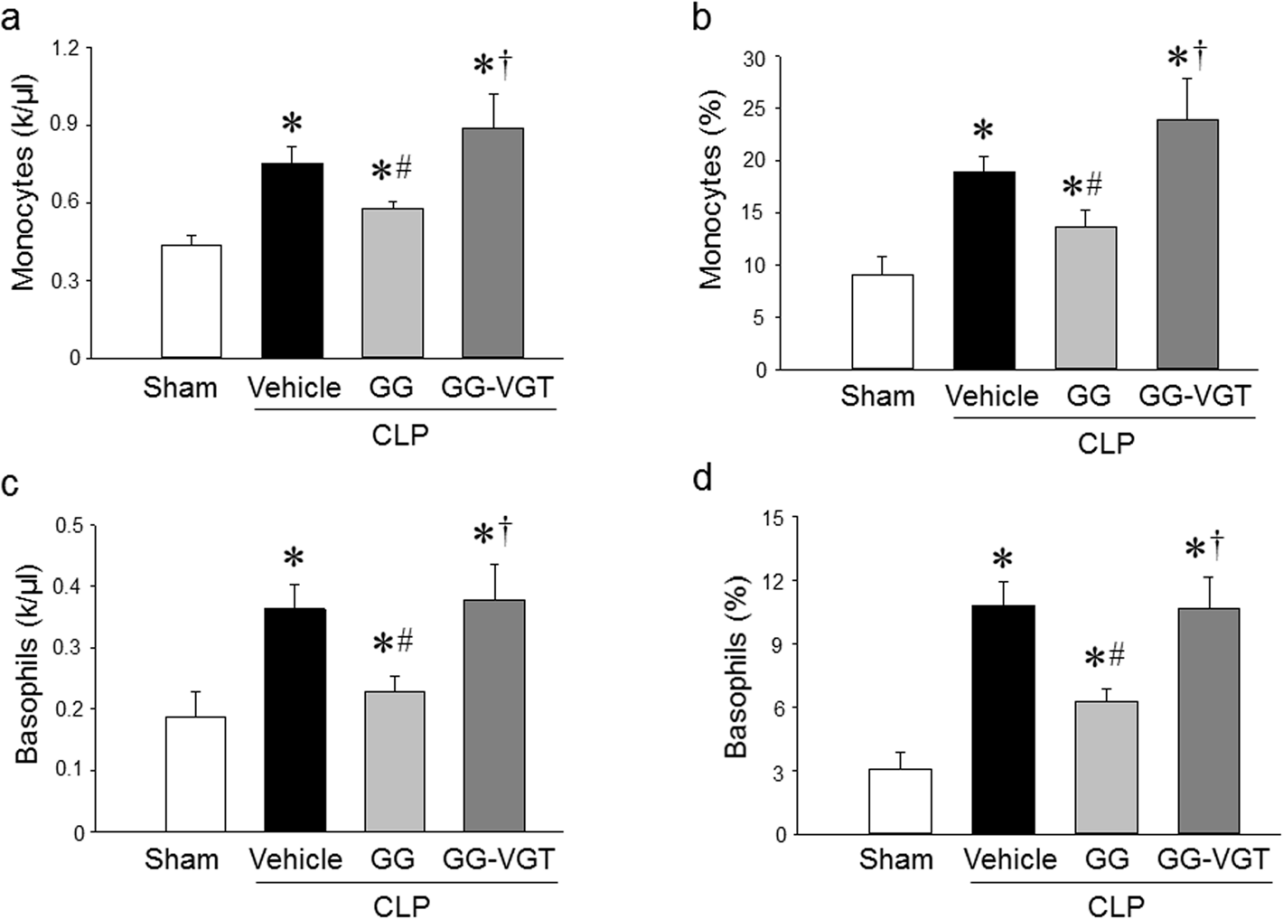
Ghrelin and GH in combination correct the levels of circulating monocytes and basophils in aged rats with sepsis. Rats were subjected to sham or CLP operation and treated with vehicle (normal saline) or GG (ghrelin 80 nmol/kg, GH 50 μg/kg) at 5 h after CLP. In an additional group of rats, vagotomy was performed at the time of CLP and treated with GG at 5 h after CLP. Blood was collected at 20 h after CLP. Circulating (**a, b**) monocytes and (**c, d**) basophils were measured using a hematology analyzer. Data are expressed as mean ± SEM (*n* = 4–6 rats/group). *P < 0.05 vs. sham; ^#^*P* < 0.05 vs. CLP with vehicle treatment; ^†^*P* < 0.05 vs. CLP with GG treatment

### Co-treatment with ghrelin and GH decreases PD-1 and restores splenic expression of HLA-DR in sepsis

Programmed death-1 (PD-1) protein expressed in immune cells negatively regulates the immune response of immune cells to antigen (Fife and Pauken 2011). We assessed the expression of PD-1 in the splenic tissue of septic rats treated with or without GG. The expression of PD-1 in the spleen of septic aged rats was upregulated at 20 h after CLP compared to aged sham rats **(**Fig. 5**)**. By contrast, treatment with GG markedly downregulated the PD-1 expression in the spleen in aged septic rats compared to vehicle-treated animals; while vagotomy reduced the effect of GG on inhibiting PD-1 expression in the spleen of aged septic rats **(**Fig. 5**)**. Human leukocyte antigen-DR (HLA-DR), a major histocompatibility complex (MHC) class II cell surface receptor expressed on antigen presenting cells like macrophages presents processed antigen to T cells to activate them (Drewry et al. 2016). We assessed the expression of HLA-DR in splenic tissues of septic rats treated with or without GG. The expression of HLA-DR in the spleen of septic aged rats was decreased at 20 h after CLP compared to aged sham rats **(**Fig. 5**)**. On the other hand, treatment with GG restored the HLA-DR expression in the spleen in aged septic rats compared to vehicle-treated animals, while vagotomy minimized the effect of GG on upregulating HLA-DR expression in the spleen of aged septic rats **(**Fig. 5**)**.

**Fig. 5:**
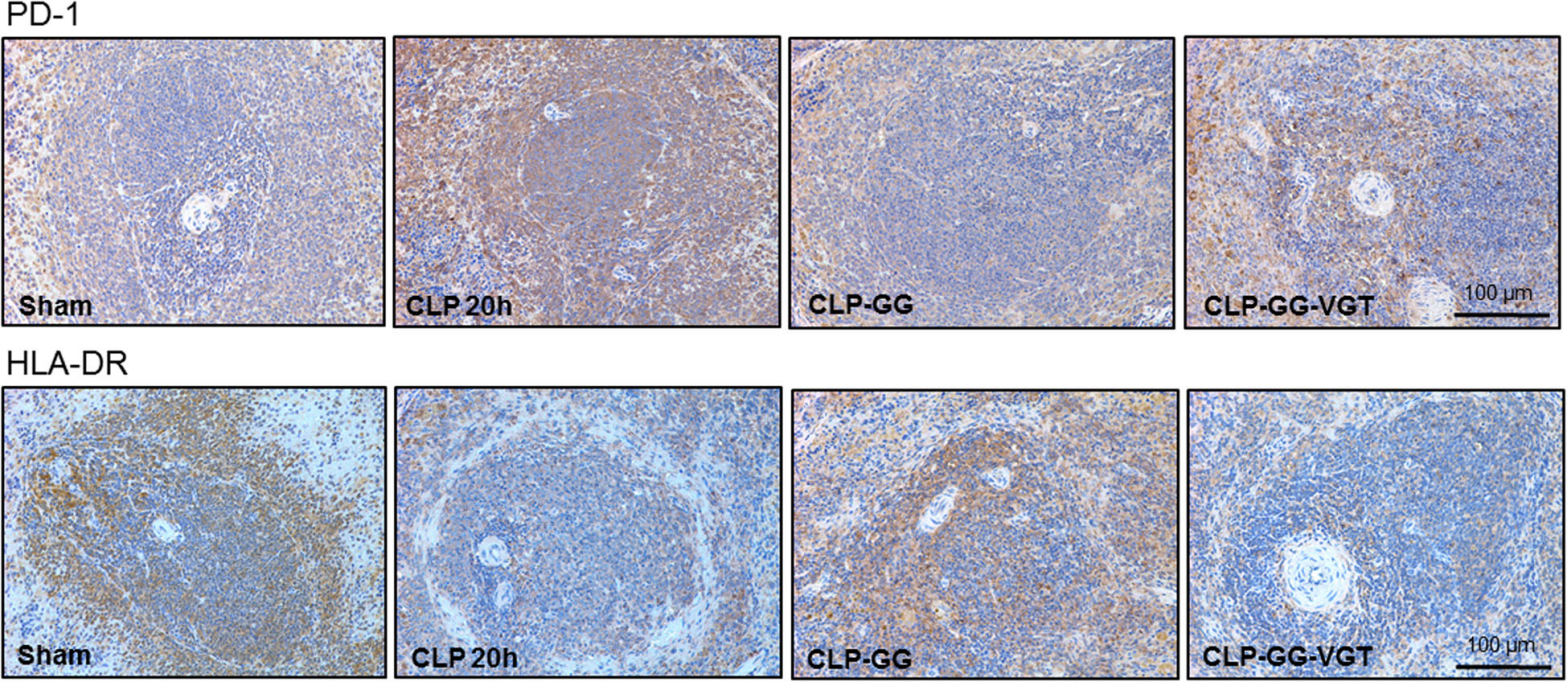
Ghrelin and GH in combination inhibit PD-1 and increase HLA-DR expression in the spleen of aged rats with sepsis. Rats were subjected to sham or CLP operation and treated with vehicle (normal saline) or GG (ghrelin 80 nmol/kg, GH 50 μg/kg) at 5 h after CLP. In an additional group of rats, vagotomy was performed at the time of CLP and treated with GG at 5 h after CLP. Spleens were harvested at 20 h after CLP. The expression of PD-1 and HLA-DR in the spleen were evaluated by immunohistochemical staining. The representative images are shown. Each group contains *n* = 4 spleens from 4 mice. Original magnification of images is 200 ×

### In vitro treatment of ghrelin and GH does not alter the immune response of splenocytes from septic aged rats

Splenocytes were isolated from sham and septic aged rats at 20 h after CLP and the cells were stimulated with LPS to induce cytokine release. Ghrelin and GH were added independently or in combination into the culture medium to determine their effect on the response of cells to LPS stimulation. As expected, the splenocytes from sham animals released significantly higher levels of TNF-α and IL-6 in response to LPS stimulation, as compared to the splenocytes isolated from septic aged rats **(**Fig. 6a, b**)**. In vitro treatment of ghrelin, GH alone, or their combination, GG, didn’t markedly increase the immune response of these splenocytes from septic aged animals **(**Fig. 6a, b**)**. In addition, ghrelin, GH alone, or GG also had no effect on cytokine release from the splenocytes of sham-operated animals **(**Fig. 6a, b**)**. These results further suggest that the increase in immune response by GG after sepsis is mediated in a vagus nerve dependent manner.

**Fig. 6:**
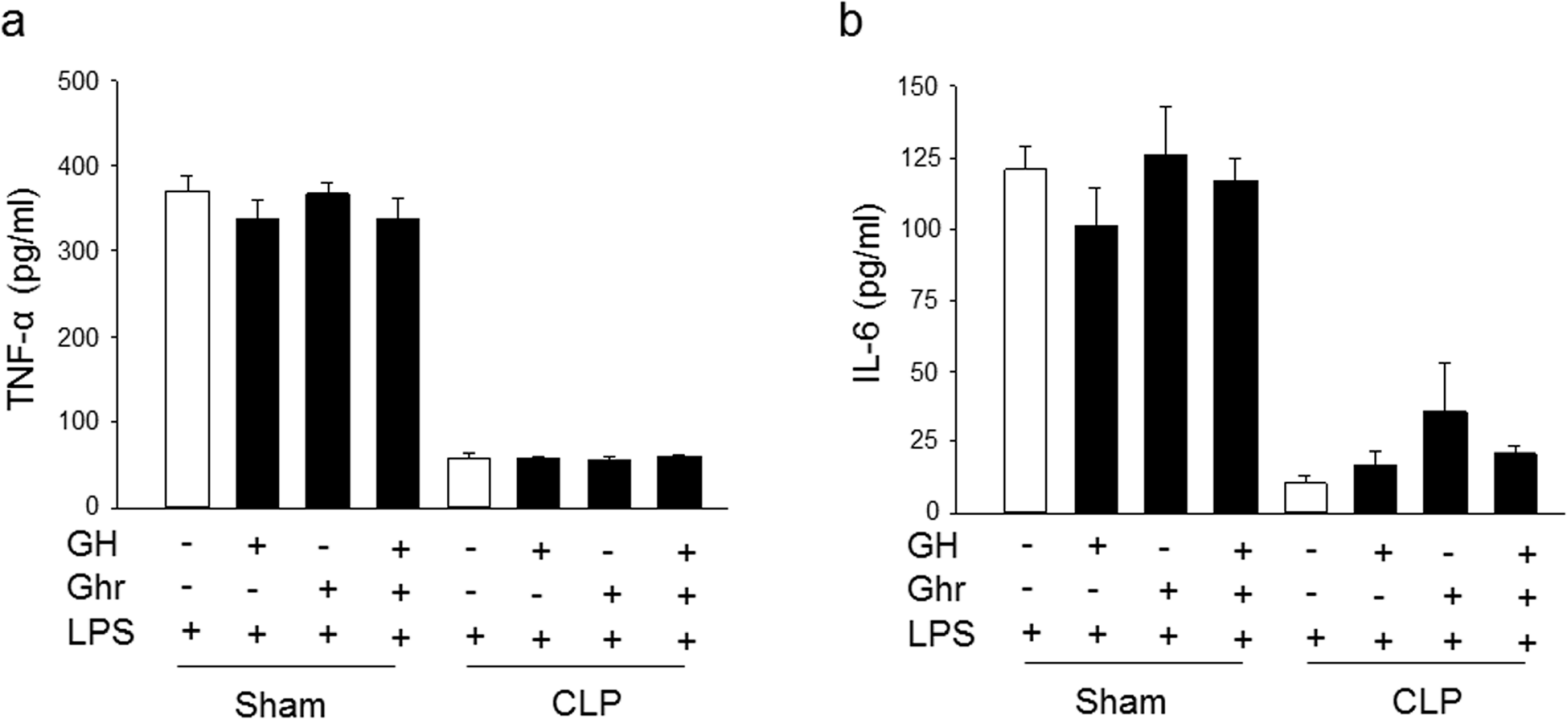
In vitro treatment of ghrelin and GH have no effect on the immune response of splenocytes of aged septic rats. Rats were subjected to sham or CLP operation. Spleens were harvested at 20 h after CLP. Splenocytes were isolated and treated with ghrelin (Ghr), GH or GG in vitro, followed by the stimulation with LPS (100 ng/ml) for 5 h. The release of (**a**) TNF-α and (**b**) IL-6 in the medium were measured by ELISA. Data are expressed as mean ± SEM (*n* = 3 rats/group)

## Discussion

In the current study, we demonstrated that TGF-β plays a critical role in sepsis-induced immunosuppression in aged septic rats. Although TGF-β has been shown to play an important role in cell growth and differentiation (Yoshimura et al. 2010; Santarpia et al. 2015; Flavell et al. 2010), subsequent study revealed its inhibitory role in lymphocyte proliferation and activation through the generation of T regulatory (Treg) cells (Wrzesinski et al. 2007). TGF-β can induce lymphocyte apoptosis in pathological conditions (Lee and Bae 2002). Tumor cells produce high levels of TGF-β that inhibit immune cells’ function to eliminate cancer cells and fosters tumor growth and metastasis (Wrzesinski et al. 2007). The role of TGF-β has been demonstrated in cancer and other diseases (Blobe et al. 2000), but its role in the development of sepsis-associated immunosuppression has not been fully revealed. Our study determined the increased levels of TGF-β in the serum and spleen, which were associated with the decreased lymphocytes, increased monocytes and basophils in the blood. We found increased expression of cleaved caspase-3 and immune co-inhibitory molecule PD-1, as well as decreased expression of HLA-DR in the spleen of aged sepsis rats. We also revealed that the splenocytes from septic aged rats exhibited a poor immune response to LPS stimulation ex vivo. We found that the combined treatment of ghrelin and GH inhibited TGF-β production, attenuated lymphopenia, corrected monocytosis and basophilia in the blood, downregulated the expression of cleaved caspase-3 and PD-1, and upregulated the expression of HLA-DR in the spleen. Similarly, the splenocytes from GG treated septic animals demonstrated a marked improvement in their immune responses to the ex vivo LPS stimulation. Moreover, our results indicate that the protective effect of GG in restoring the lymphocyte contents and their immune response was mediated through the vagus nerve. We previously demonstrated that treatment with ghrelin alone protects young animals from sepsis, but not aged animals (Wu et al. 2009a). Ghrelin or GH alone does not show protective effect in aged animals because the sensitivity and expression of ghrelin receptors are reduced in aged rats compared to young rats (Wu et al. 2009a). Treatment with a low dose of GH increases the sensitivity and expression of ghrelin receptors. Therefore, the combined treatment with ghrelin and GH shows protection in aged septic animals (Wu et al. 2009a).

Sepsis induced apoptosis causes profound depletion of B and CD4^+^ T lymphocytes in humans (Hotchkiss et al. 2001). Immune cell apoptosis in sepsis mainly occurs through the caspase-mediated pathway (Aziz et al. 2014). We previously showed that GG treatment attenuates lymphopenia and restore the loss of CD4^+^ and CD8^+^ T cells in sepsis by inhibiting the expression of cleaved caspase-3 and caspase-8 (Yang et al. 2016; Zhou et al. 2010; Zhou et al. 2017). In the current study, we revealed that the GG treatment attenuates lymphopenia in sepsis by inhibiting the expression of cleaved caspase-3 in the spleen.

PD-1, a co-inhibitory receptor of lymphocyte signal transduction is expressed by T and B cells and macrophages (Keir et al. 2008; Bally et al. 2015). HLA-DR is a marker of antigen presenting cells, which is mainly expressed in the professional antigen presenting cells like macrophages, dendritic cells, and B cells (Roche and Furuta 2015). The immunohistochemical staining showed that PD-1 positive cells are mainly localized at the follicles and marginal zone of while pulp of the spleen. We also found that HLA-DR positive cells were mainly localized in the marginal zone of the spleen. Our previous reports revealed that sympathetic and parasympathetic nerves are involved in the immune function in sepsis and GG treatment modulates immune responses through the vagus nerve (Zhou et al. 2017; Wu et al. 2007b). Since vagotomy without ghrelin treatment has no effect on cytokine production in septic rats compared to non-vagotomized septic rats (Wu et al. 2007b), it is likely that the vagotomy may not alter PD-1 and HLA-DR expression in septic aged rats. However, an intact vagus nerve is needed for the beneficial effects of ghrelin to attenuate the dysregulated immune responses (Wu et al. 2007b). Vagotomy blocked the effect of GG on decreasing PD-1 and increasing HLA-DR expression in the spleen of aged septic rats. Tregs are increased in the spleen during sepsis (Zhou et al. 2017). We previously showed that GG treatment reduced Tregs which contributed to the beneficial effect of GG on attenuation of immunosuppression (Zhou et al. 2017). We also revealed that vagotomy blocked the beneficial effect of GG on reducing the contents of Tregs in aged septic rats. PD-1 promotes Treg development and survival (Francisco et al. 2009). Since PD-1 expression was decreased in aged sepsis rats after treatment with GG, it is reasonable that the GG-mediated decrease in Treg population could be due to the decreased expression of PD-1 in lymphocytes.

Sepsis-induced immunosuppression results from the loss of lymphocytes, increasing the expression of co-inhibitory molecules, and the differentiation of Treg (Hotchkiss et al. 2013; Boomer et al. 2011; Hotchkiss and Karl 2003). These alterations can be linked to increased TGF-β production. Elevated levels of TGF-β have been reported in patients with sepsis (Marie et al. 1996; Wu et al. 2009b). High levels of circulating TGF-β during sepsis caused by pneumonia have been shown to correlate with higher tissue injury scores and mortality (Wu et al. 2009b). Increased levels of TGF-β are associated with the reduced bacterial clearance and increased organ injury (Weehuizen et al. 2012). It has been shown that bacteria-induced immunosuppression is mediated through the induction of TGF-β, followed by the decrease in the expression of surface MHCII on the dendritic cells (DC), thereby inhibiting DC activation (Bosio et al. 2007). TGF-β plays a prominent role in the differentiation of immunoparalyzed DC, leading to immunosuppression after lung infection by *E. coli* (Roquilly et al. 2017). TGF-β can suppress the release of proinflammatory mediators such as TNF-α and IL-1β from monocytes and macrophage (Blobe et al. 2000; Pellacani et al. 2001). Patients with sepsis can have both increased or decreased levels of TGF-β (Marie et al. 1996; Pellacani et al. 2001; White et al. 2010), which could be due to the variant stages of sepsis in these patients.

In sepsis, the host response to infection is associated with sustained inflammation to eliminate pathogens in the early phase and immune suppression in the later phase (van der Poll et al. 2017). Patients with sepsis show signs of both excessive inflammation and immune suppression, although the extent of which may vary between individuals (van der Poll et al. 2017). TGF-β signaling is important to maintain immune homeostasis in sepsis and other infectious conditions. The dysregulation of TGF-β has been associated with tissue injury and higher mortality in sepsis (Wu et al. 2009b; Lekkou et al. 2014). Sepsis survivors have been found to have higher levels of TGF-β in early time points and lower levels of TGF-β at 10 days after hospitalization (Lekkou et al. 2014). In contrast, the non-survivors have lower levels of TGF-β in early stages and higher levels of TGF-β at 10 days after their admission in the hospital (Lekkou et al. 2014). Elderly are particularly susceptible to infections due to insufficient immunity. Aged septic patients have features consistent with immunosuppression that result in an inability to clear infections and provide a predisposition to developing nosocomial infections (Hotchkiss et al. 2013; Boomer et al. 2011; Hotchkiss and Karl 2003). The gene expression profile showed that TGF-β expression in elderly septic patients is higher as compared to young septic patients (Vieira da Silva Pellegrina D, et al. 2015). The higher TGF-β in elderly septic patients correlates with their suppressed immune response, higher morbidity, and mortality (Hotchkiss et al. 2013; Boomer et al. 2011). Immunosuppression was observed in aged rats at 20 h after CLP. These animals demonstrated apoptosis in T cells, an increase of co-inhibitory molecules, and Treg differentiation (Zhou et al. 2017). Our current study demonstrates the upregulation of TGF-β is associated with the immunosuppression in aged animal with sepsis. It has been shown that immunosuppression occurs at a much later time point after sepsis onset in young animals (Unsinger et al. 2012). Although the circulating TGF-β was increased in aged septic rats at 20 h after CLP, this increase was not observed in young rats at 3 months of age 20 h after CLP (data not shown). Thus, the levels of TGF-β between young and aged animals reflect the differentiated immune response between different age groups during sepsis.

TGF-β can be used as a prognostic marker of sepsis-induced immunosuppression in the elderly population. Inhibition of TGF-β has been shown to ameliorate organ injury and improve survival in sepsis (Weehuizen et al. 2012; Bae et al. 2014). Blockade of TGF-β attenuated immunosuppression and reduced the susceptibility to secondary infection following sepsis (Roquilly et al. 2017). Thus, modulating TGF-β could be a potential therapeutic target for elderly patients with sepsis. Administration of ghrelin was found to reduce TGF-β expression and exert an anti-fibrotic effect in patients with systemic sclerosis (Ota et al. 2013). Here, we demonstrated that ghrelin reduced TGF-β levels in the serum in septic aged animals and ameliorated sepsis-induced immune suppression. Human ghrelin has been administered into human subjects in clinical studies, showing excellent efficacy and safety profile (Narula and deBoisblanc 2015). Ghrelin has 93% homology in its amino acid sequence between human and rat or human and mouse (Kojima and Kangawa 2005). In the current study, we used human ghrelin as a clinically relevant approach for implementing ghrelin as a novel therapeutic for elderly septic patients.

Communication between the nervous and immune systems is important for the regulation of immune function and inflammation (Tracey 2007; Abe and Inoue 2018; Inoue et al. 2016). The vagus nerve has been shown to play a critical role in the inflammatory reflex and mediates a rapid homeostatic response to protect against organ injury (Tracey 2007; Abe and Inoue 2018; Inoue et al. 2016). The anti-inflammatory effect of ghrelin is mainly mediated through the vagus nerve as vagotomy diminishes the protective effect of ghrelin on septic animals (Wu et al. 2007b). The afferent vagus nerve can be activated by peripheral inflammatory stimulation and the signal is transmitted to the efferent vagus nerve to suppress the inflammation (Abe and Inoue 2018; Borsody and Weiss 2005). The activation of the vagus nerve during infection results in a protective advantage to the host, while a defect with the anti-inflammatory pathway of the vagus nerve contributes to disease pathology (Tracey 2007; Tracey 2005). In addition, the immunomodulatory effect of the efferent vagal nerve on macrophages is mediated through the stimulation of cholinergic activity of the parasympathetic nervous system (Tracey 2007). The spleen is a primary organ of the cholinergic anti-inflammatory pathway (Tracey 2007; Inoue et al. 2016). The impaired cholinergic anti-inflammatory pathway in aged rats is responsible for the robust inflammatory response after the onset of sepsis resulting in early morbidity and mortality in aged septic animals (Wu et al. 2009a). On the other hand, aged animals that survived sepsis showed an impaired immune response to LPS stimulation (Zhou et al. 2017). This indicates that these aged animals cannot remove bacteria efficiently during secondary infection, which may lead to higher mortality rates after sepsis (Wu et al. 2009a; Yang et al. 2016). Despite the anti-inflammatory activity of the vagus nerve, activation of the immune response has been shown to occur in immune dysregulated conditions (Corcoran et al. 2005). Vagus nerve stimulation has been shown to increases immune activity by elevating plasma levels of inflammatory cytokines, such as TNF-α and IL-6 in humans with depression (Corcoran et al. 2005). Depression has been characterized as a dysregulation of immune function and has been associated with immunosuppression (Blume et al. 2011; Zhang et al. 2018).

## Conclusions

In conclusion, we identified that GG maintained the immune response in aged septic rats through the inhibition of TGF-β via vagal stimulation. The decreased expression of TGF-β improved the immune response to infection in aged septic rats by maintaining lymphocyte counts, correcting monocytosis and basophilia that correlated with the decreased expression of cleaved caspase-3, reducing lymphocyte inhibitory receptor PD-1, and increasing HLA-DR expression.

## Data Availability

The manuscript does not contain any supplemental or supportive data file.

## Abbreviations

CLP: Cecal ligation and puncture
GG: Ghrelin and growth hormone in combination
GH: Growth hormone
GHSR: Growth hormone secretagogue receptor
PD-1: Programmed death-1
TGF-β: Transforming growth factor-β
Treg: T regulatory cells
